# Supplementary material for: Tensor-Decomposition-Based Unsupervised Feature Extraction Applied to Prostate Cancer Multiomics Data
Source: Genes (Basel). 2020 Dec 11;11(12):1493. doi: 10.3390/genes11121493 (PMC7763286; doi:10.3390/genes11121493)
Supplement: Supplementary file 1 [file genes-11-01493-s001.zip › AnalysisReport.pptx]

## Slide 1
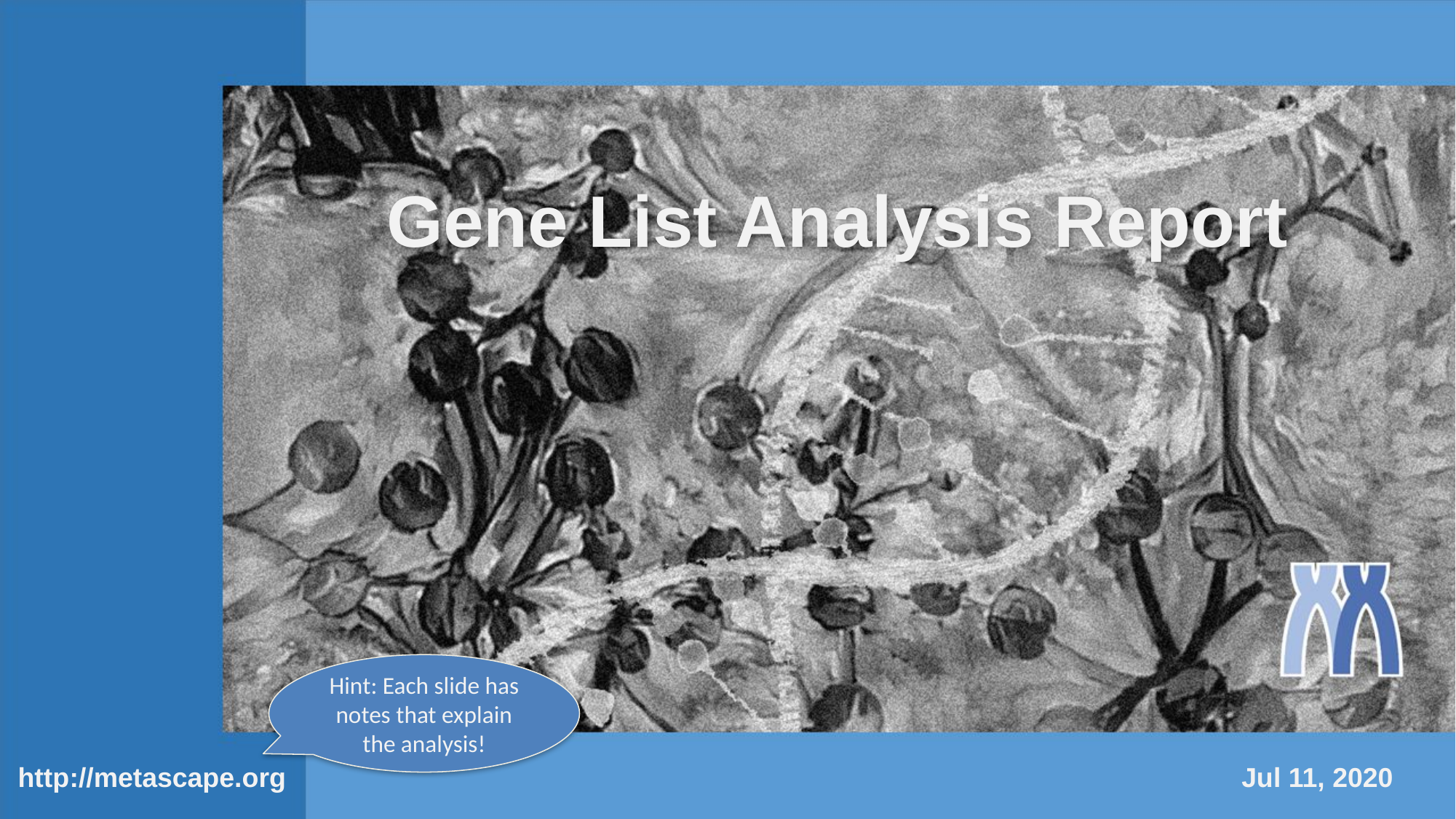

# Gene List Analysis Report
Hint: Each slide has notes that explain the analysis!
http://metascape.org
Jul 11, 2020

## Slide 2
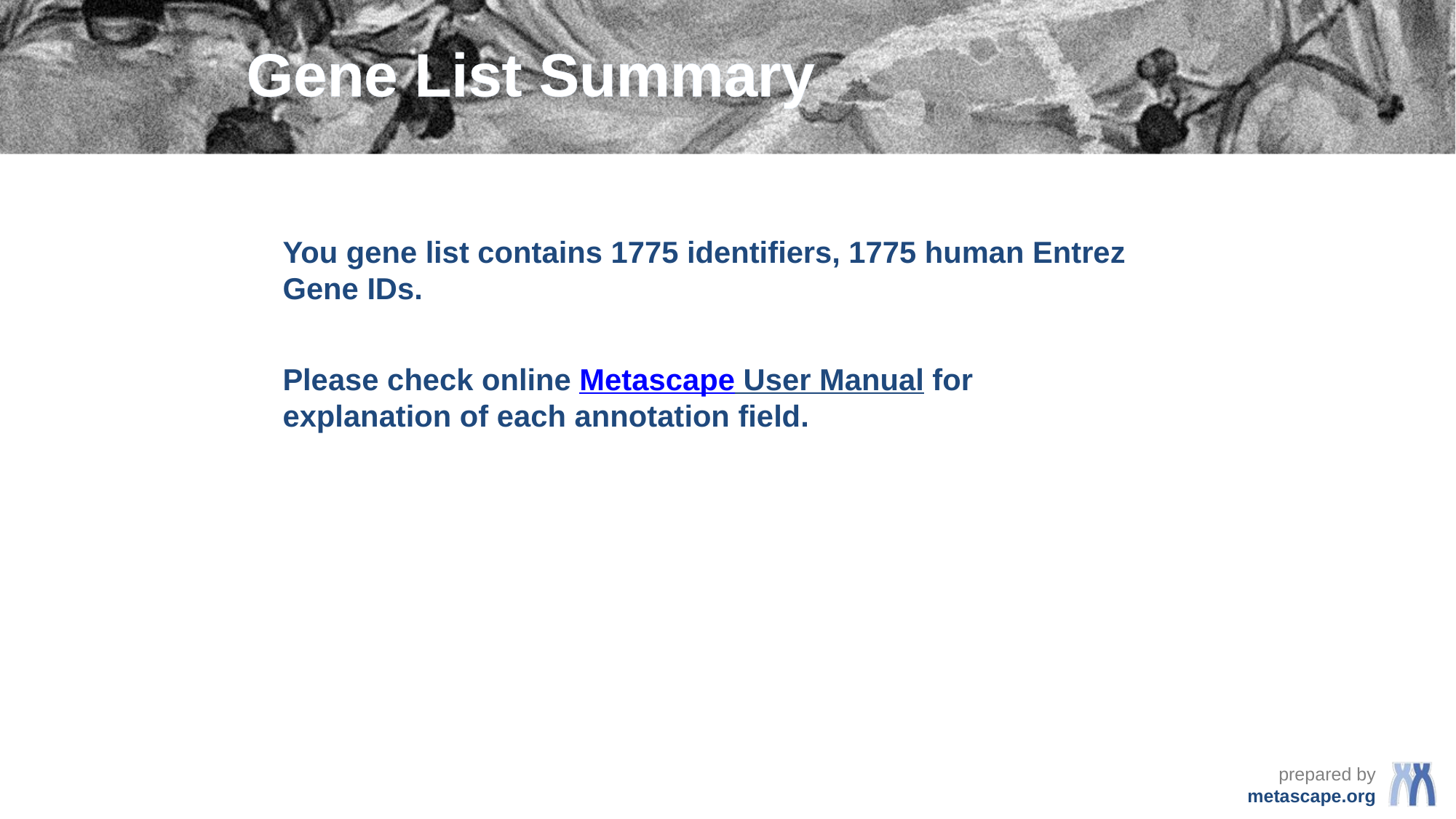

# Gene List Summary
You gene list contains 1775 identifiers, 1775 human Entrez Gene IDs.
Please check online Metascape User Manual for explanation of each annotation field.

## Slide 3
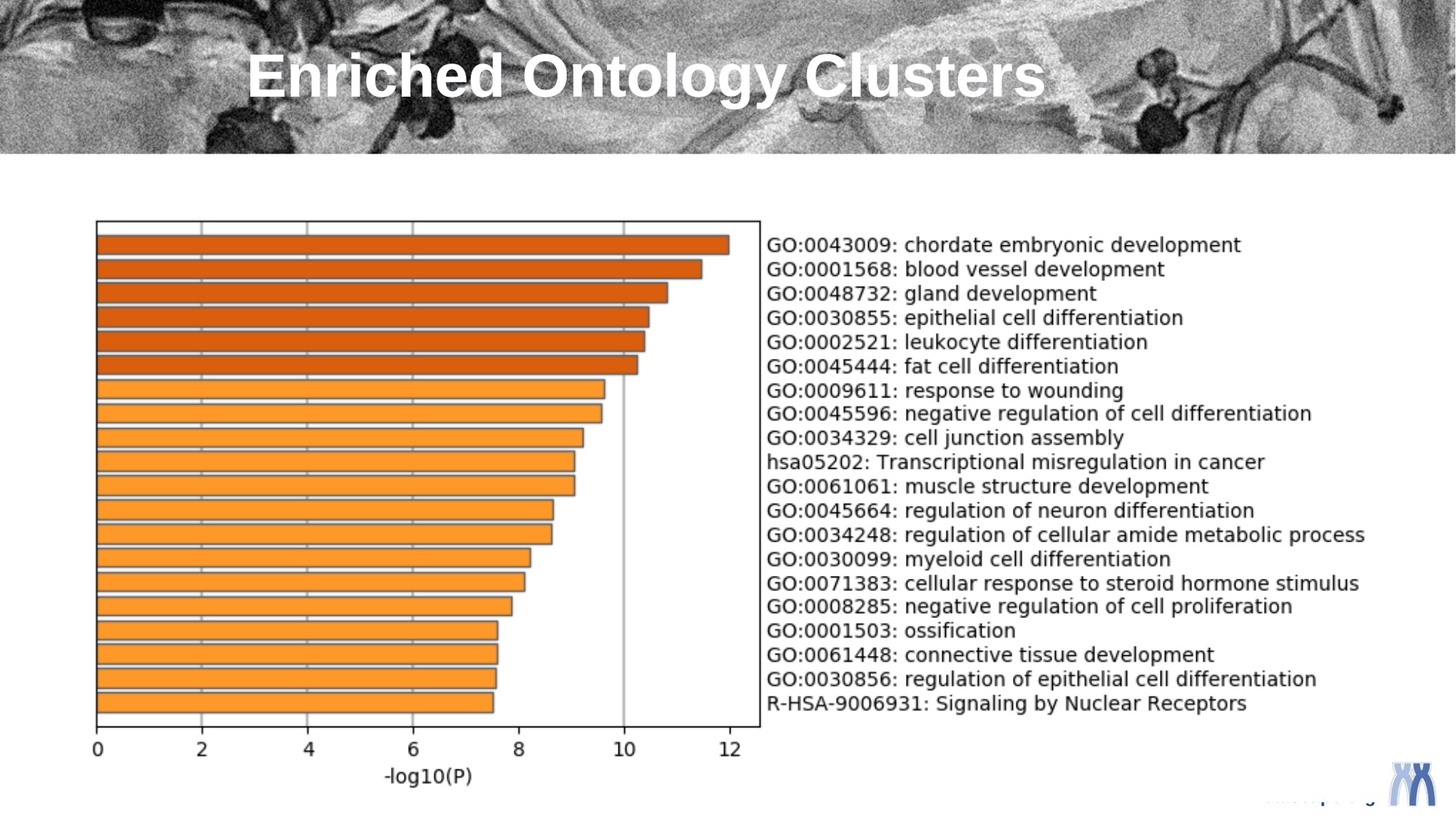

# Enriched Ontology Clusters

## Slide 4
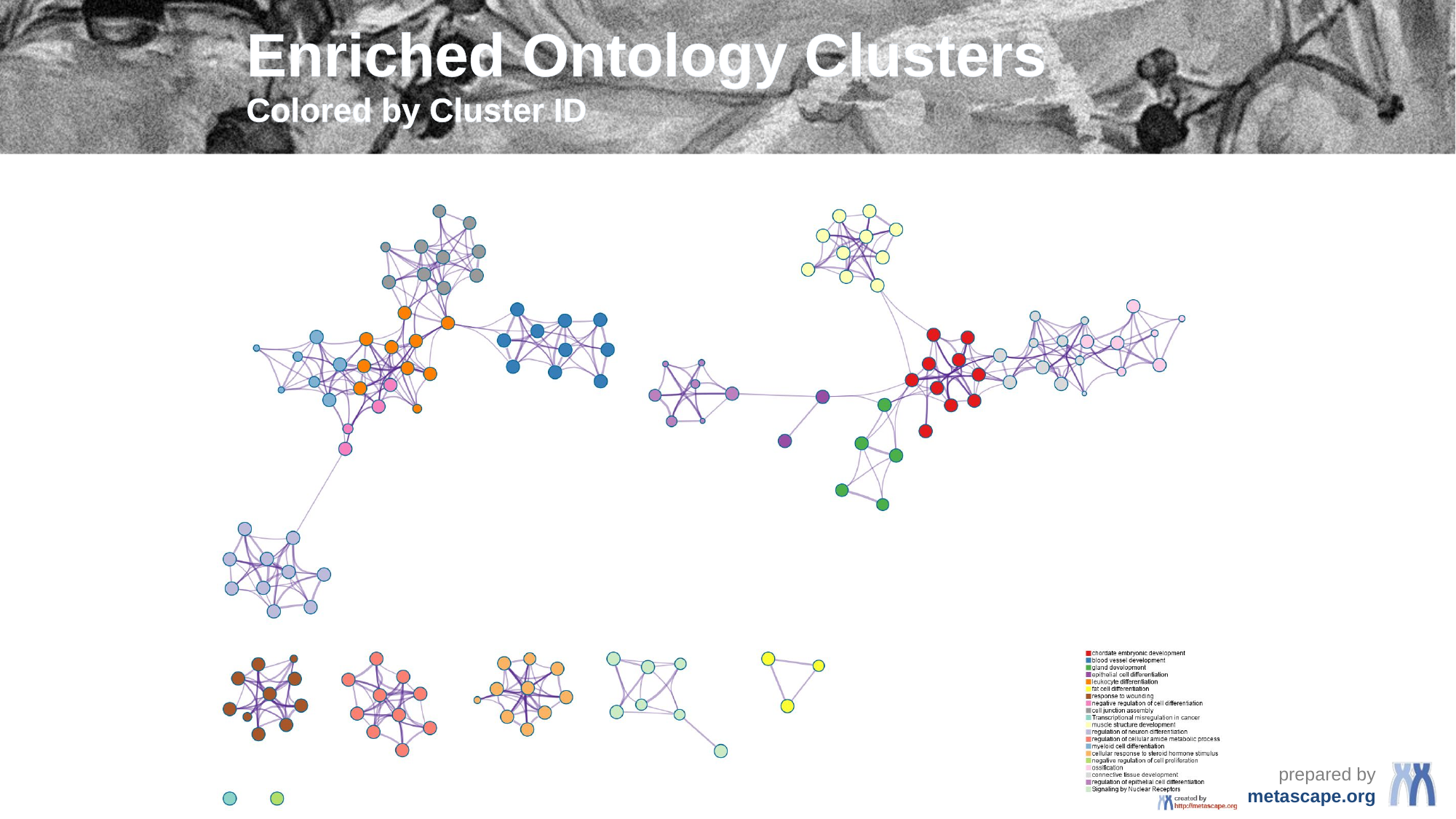

# Enriched Ontology Clusters Colored by Cluster ID

## Slide 5
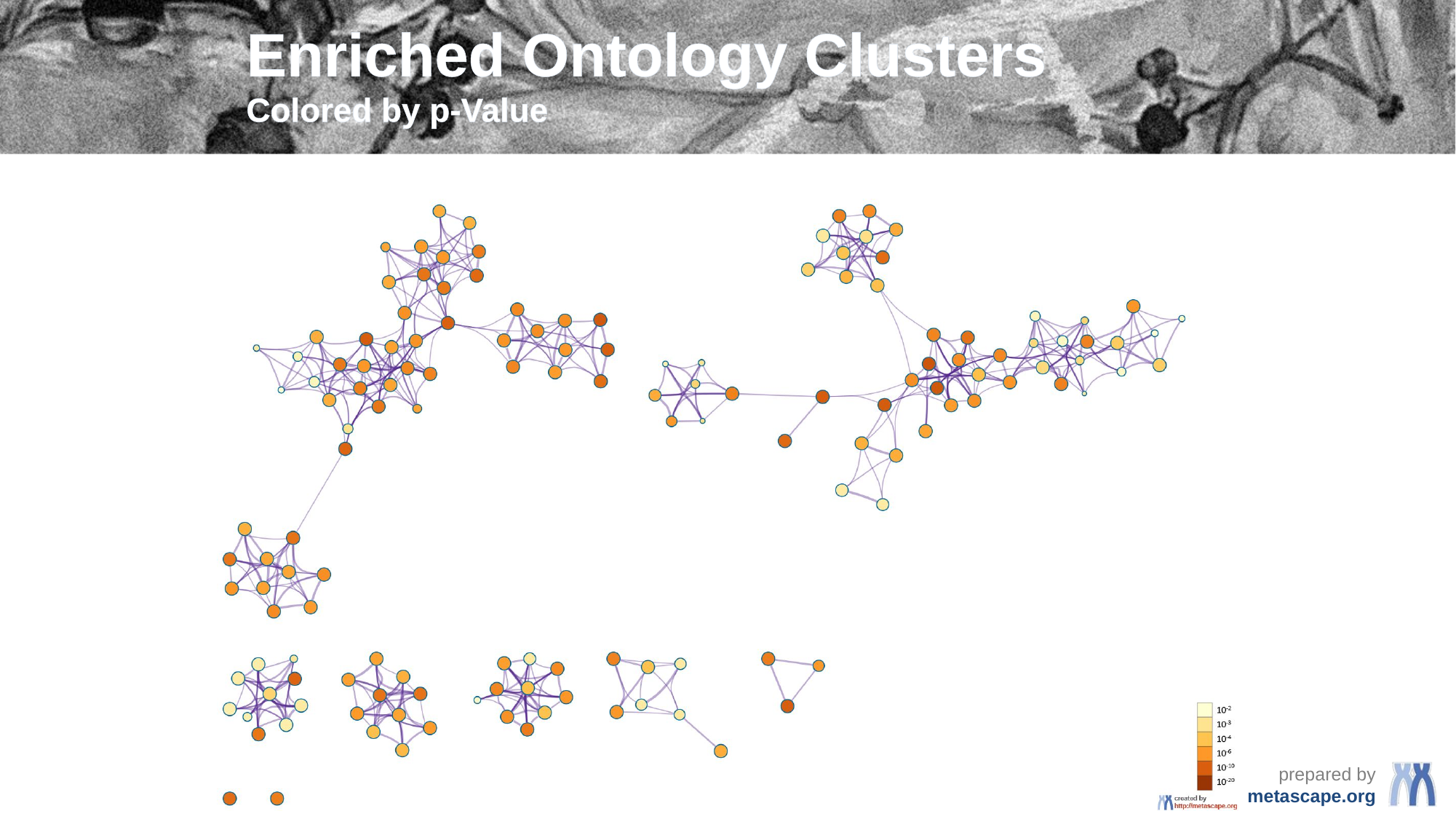

# Enriched Ontology Clusters Colored by p-Value

## Slide 6
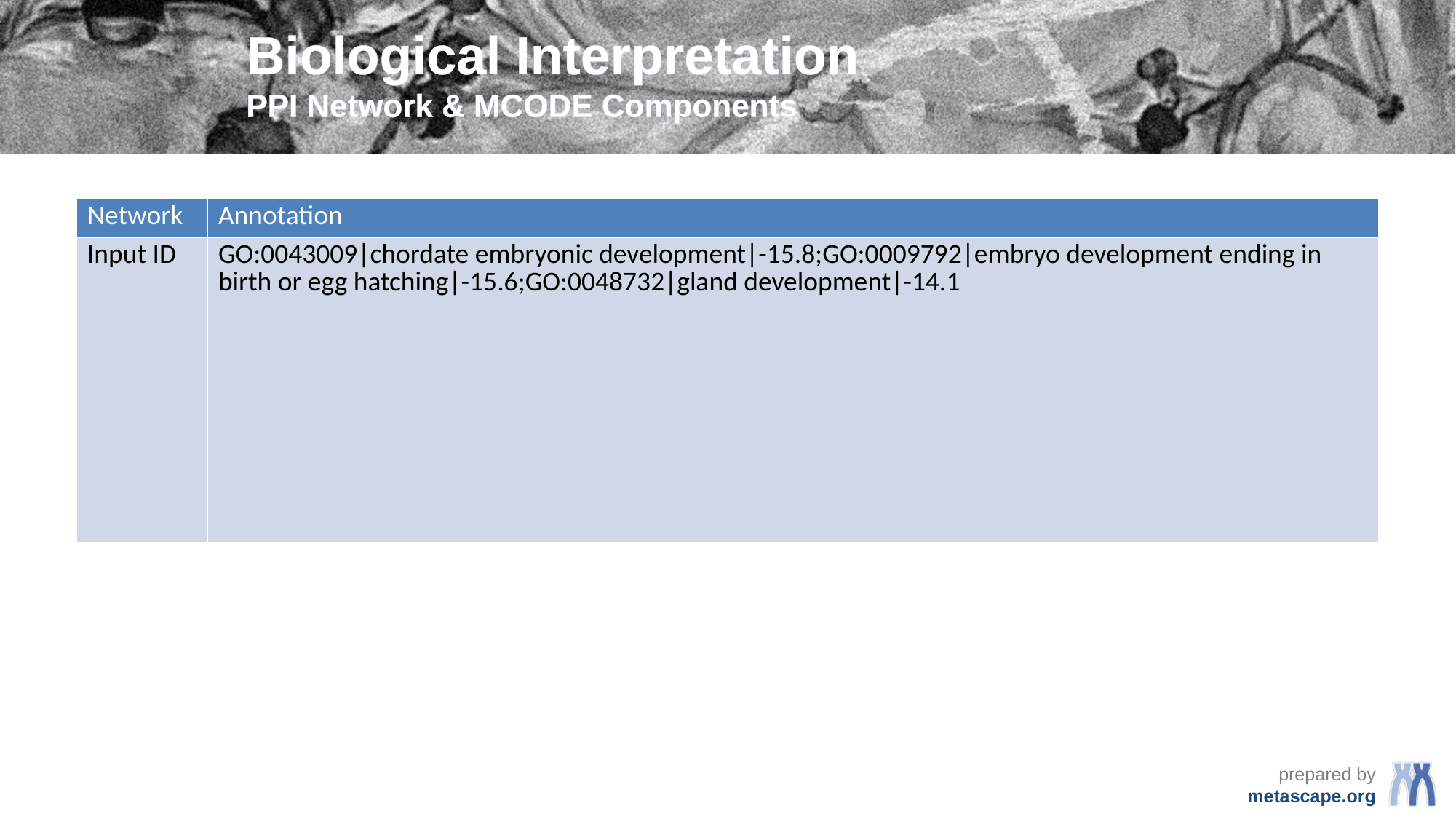

# Biological Interpretation PPI Network & MCODE Components
| Network | Annotation |
| --- | --- |
| Input ID | GO:0043009|chordate embryonic development|-15.8;GO:0009792|embryo development ending in birth or egg hatching|-15.6;GO:0048732|gland development|-14.1 |

## Slide 7
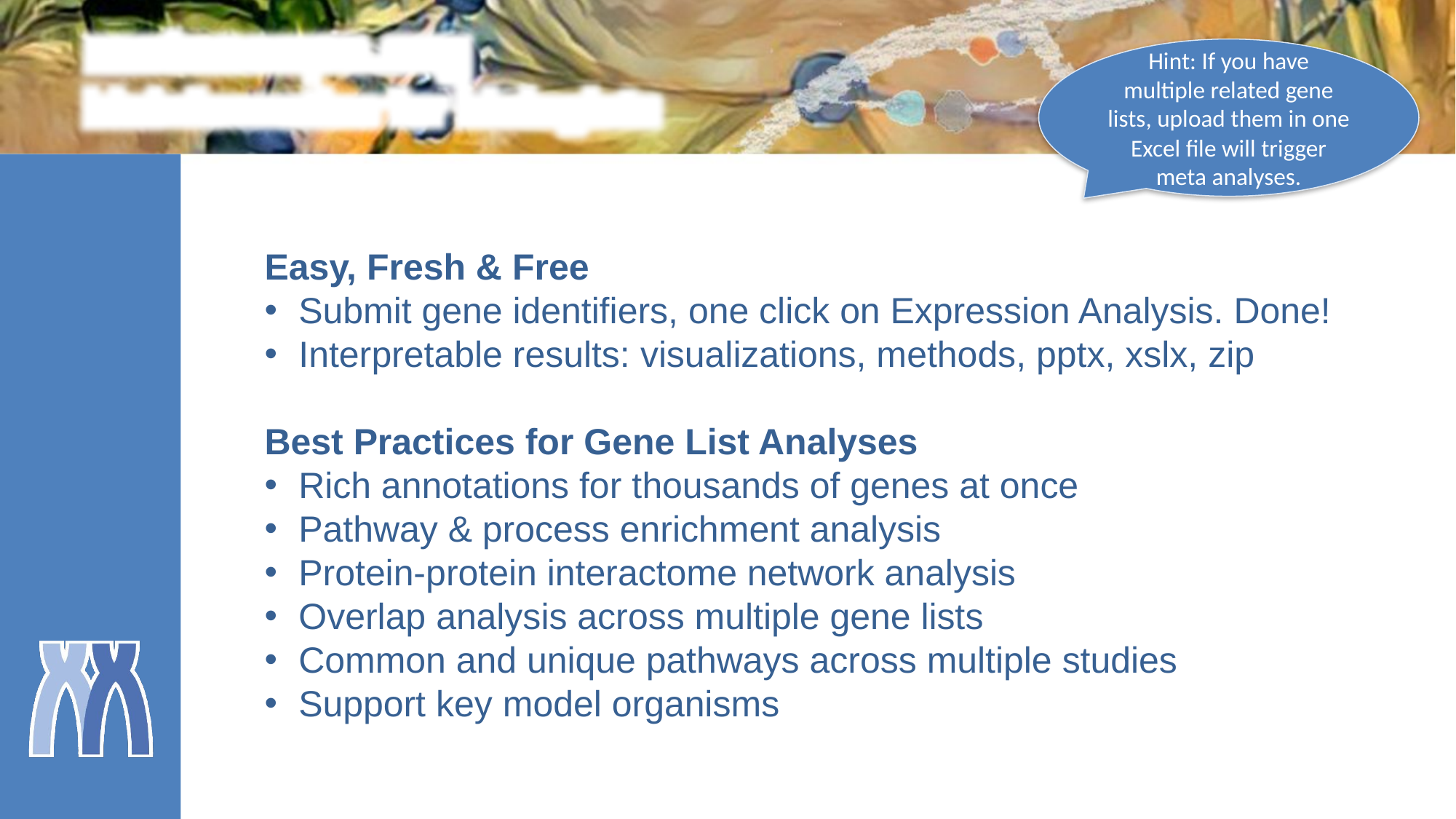

metascape.org
bioinformatics for biologists
Hint: If you have multiple related gene lists, upload them in one Excel file will trigger meta analyses.
Easy, Fresh & Free
Submit gene identifiers, one click on Expression Analysis. Done!
Interpretable results: visualizations, methods, pptx, xslx, zip
Best Practices for Gene List Analyses
Rich annotations for thousands of genes at once
Pathway & process enrichment analysis
Protein-protein interactome network analysis
Overlap analysis across multiple gene lists
Common and unique pathways across multiple studies
Support key model organisms
