# Supplementary figures and images for: Tensor-Decomposition-Based Unsupervised Feature Extraction Applied to Prostate Cancer Multiomics Data
Source: Genes (Basel). 2020 Dec 11;11(12):1493. doi: 10.3390/genes11121493 (PMC7763286; doi:10.3390/genes11121493)

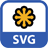

Supplement: Supplementary file 1 [file genes-11-01493-s001.zip › icon/SVG48.png]

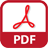

Supplement: Supplementary file 1 [file genes-11-01493-s001.zip › icon/PDF48.png]

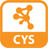

Supplement: Supplementary file 1 [file genes-11-01493-s001.zip › icon/CYS48.png]

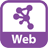

Supplement: Supplementary file 1 [file genes-11-01493-s001.zip › icon/WEB_CYS48.png]

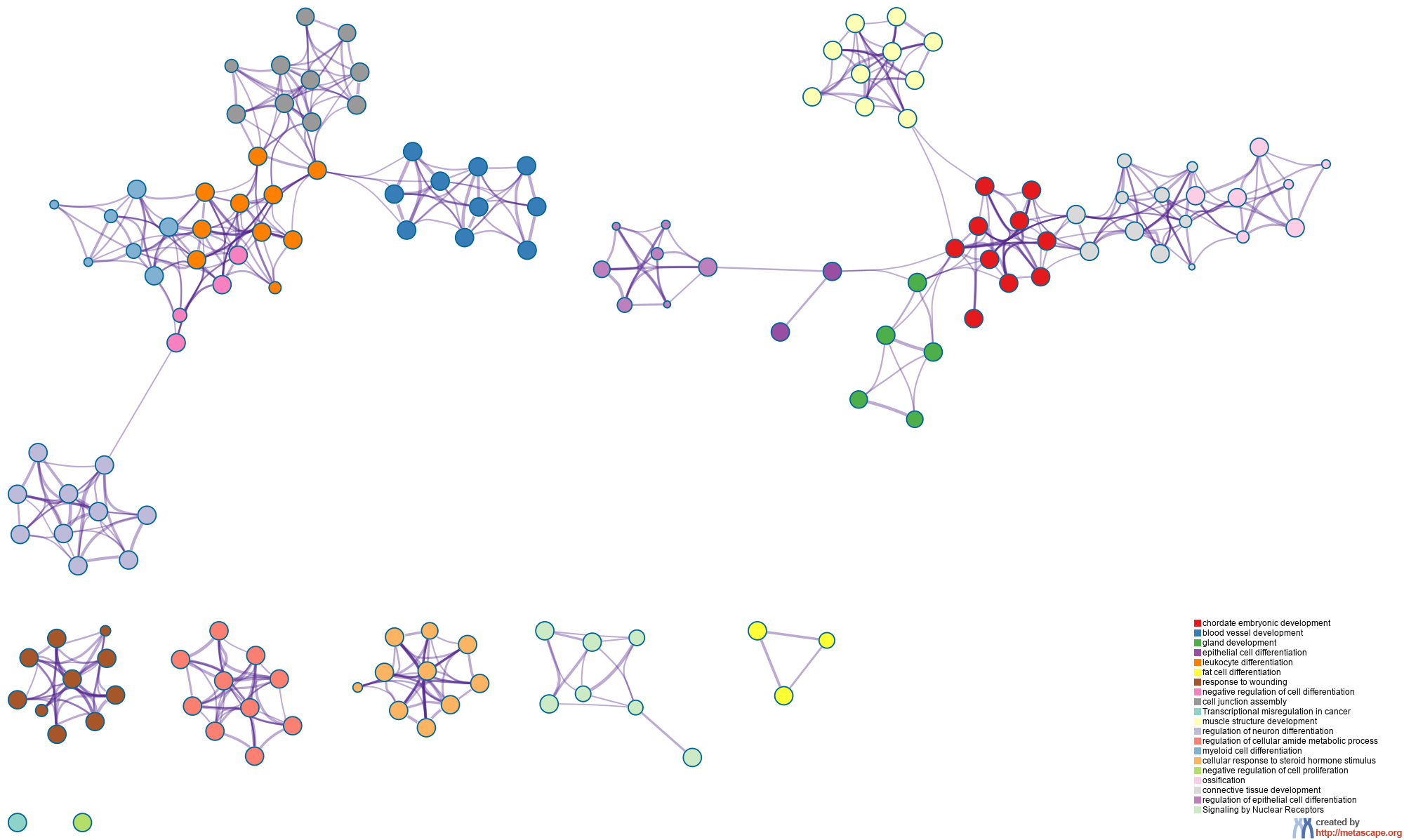

Supplement: Supplementary file 1 [file genes-11-01493-s001.zip › Enrichment_GO/ColorByCluster.png]

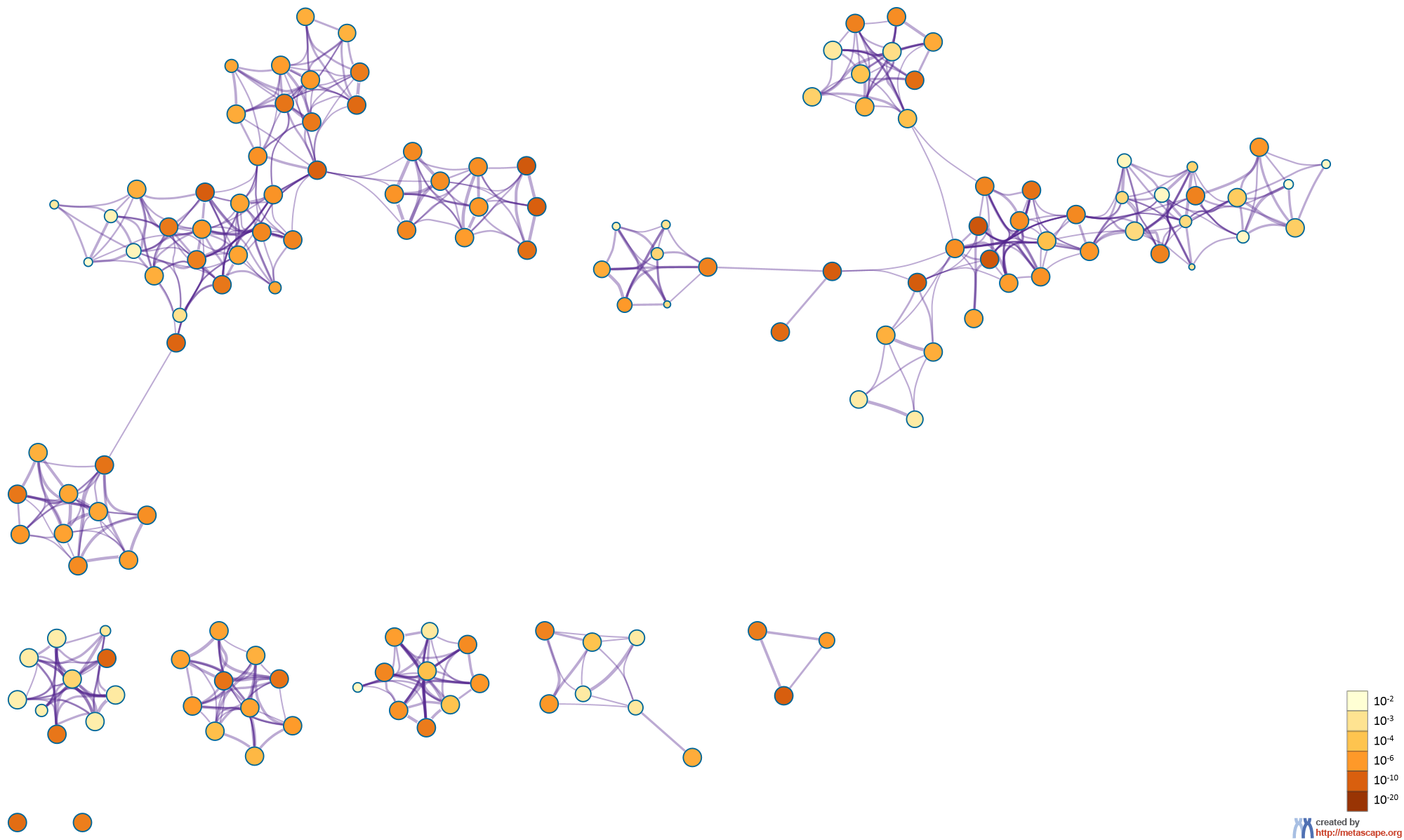

Supplement: Supplementary file 1 [file genes-11-01493-s001.zip › Enrichment_GO/ColorByPValue.png]

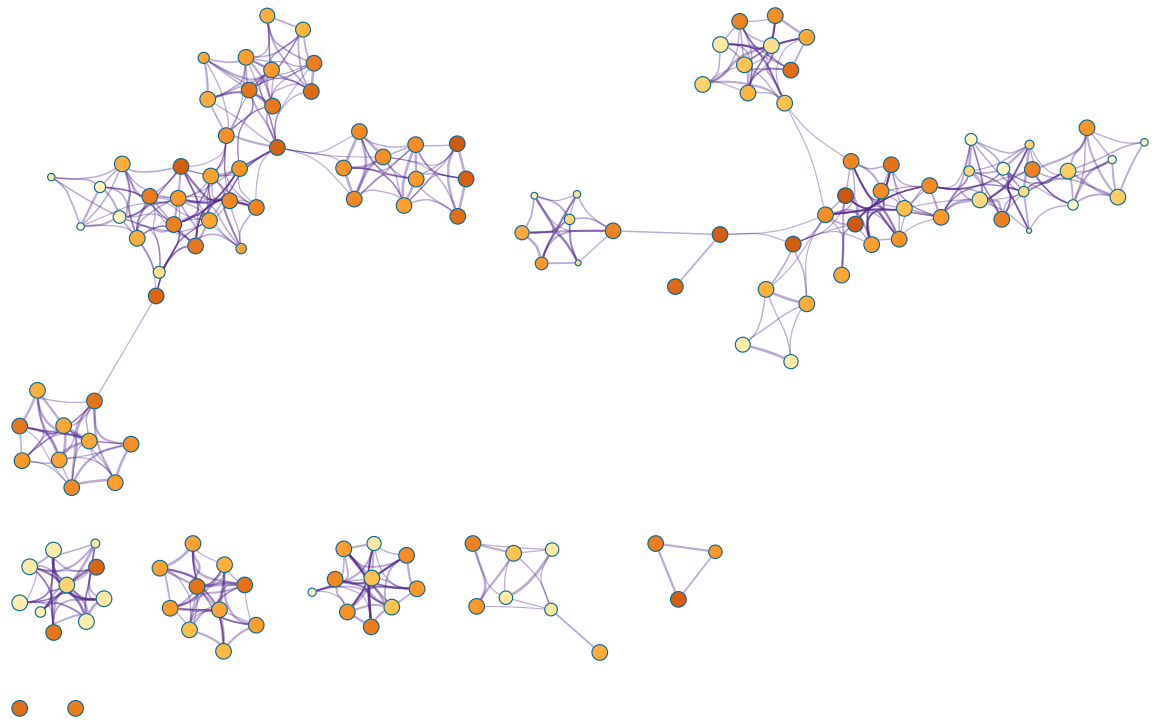

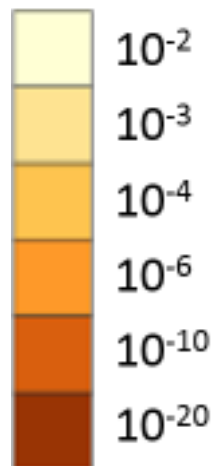

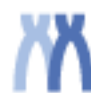 created by  
<http://metascape.org>

Supplement: Supplementary file 1 [file genes-11-01493-s001.zip › Enrichment_GO/ColorByPValue.pdf]

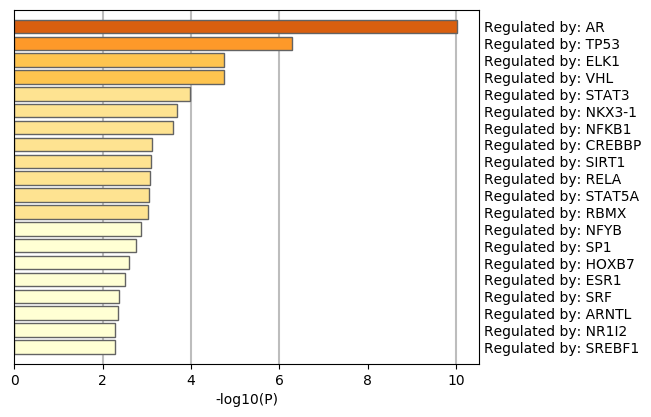

Supplement: Supplementary file 1 [file genes-11-01493-s001.zip › Enrichment_QC/HeatmapSelectedGO_TRRUST.png]

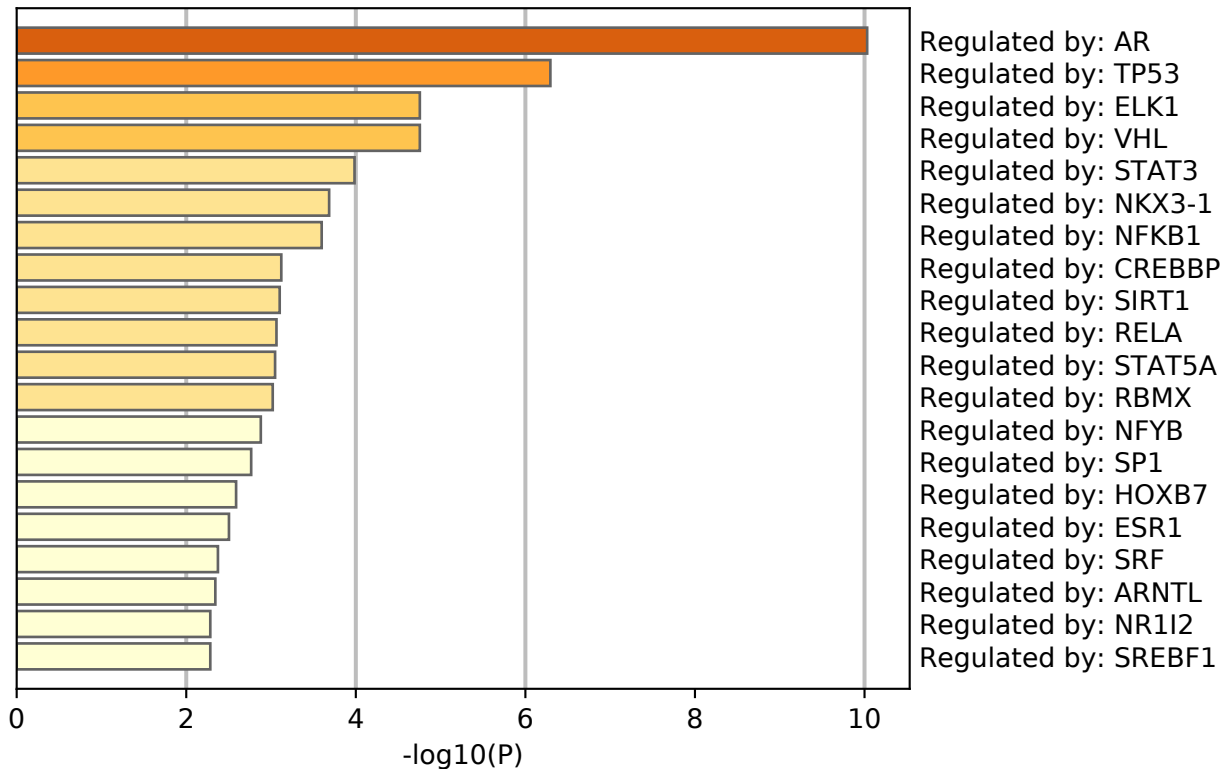

Supplement: Supplementary file 1 [file genes-11-01493-s001.zip › Enrichment_QC/HeatmapSelectedGO_TRRUST.pdf]

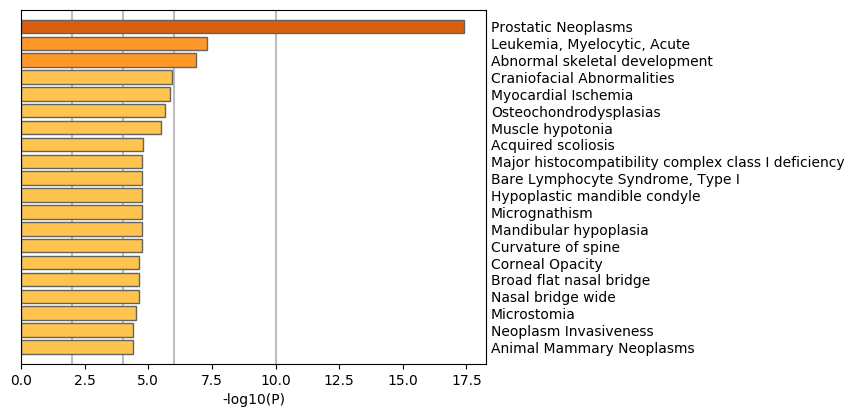

Supplement: Supplementary file 1 [file genes-11-01493-s001.zip › Enrichment_QC/HeatmapSelectedGO_DisGeNET.png]

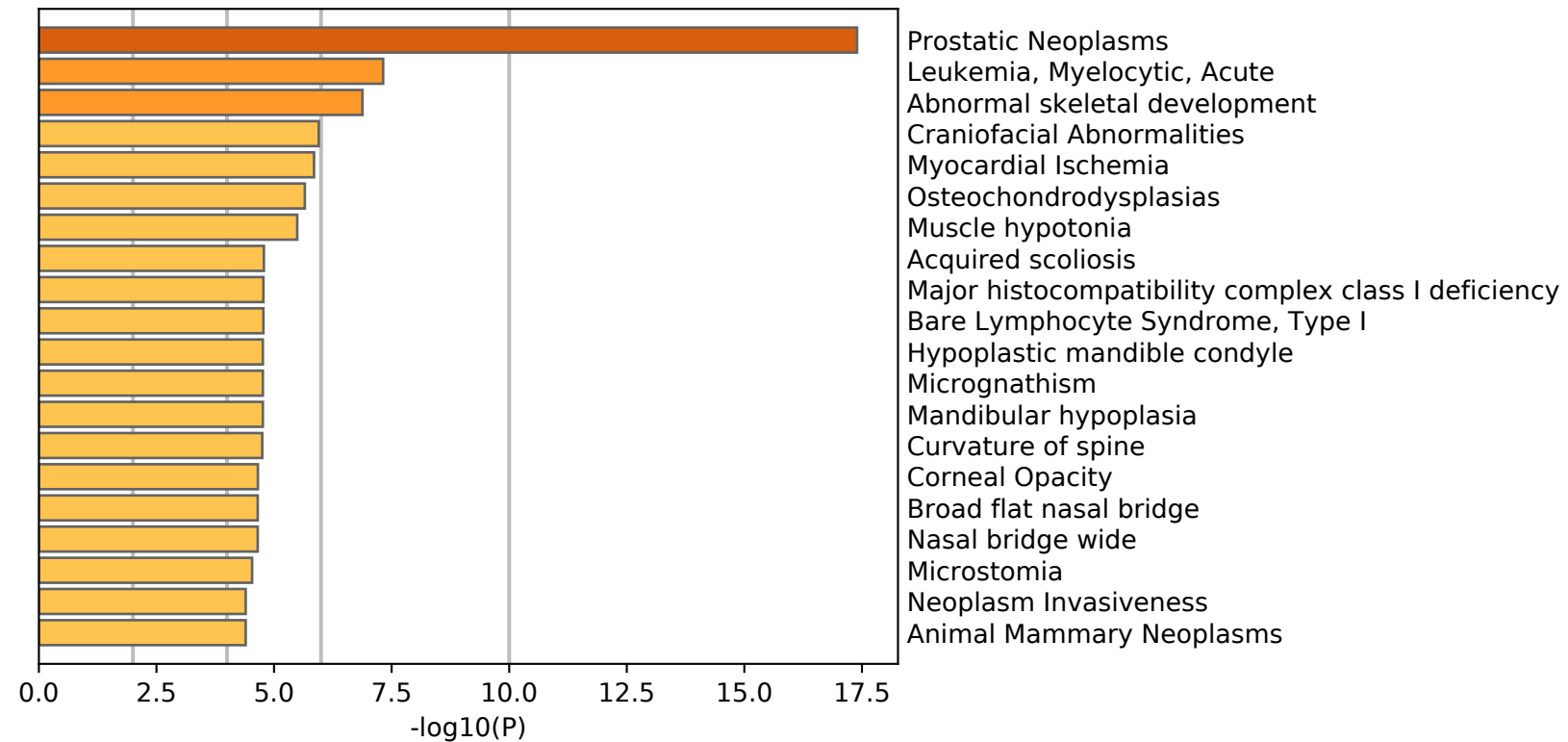

Supplement: Supplementary file 1 [file genes-11-01493-s001.zip › Enrichment_QC/HeatmapSelectedGO_DisGeNET.pdf]

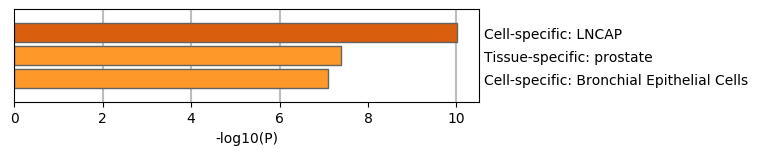

Supplement: Supplementary file 1 [file genes-11-01493-s001.zip › Enrichment_QC/HeatmapSelectedGO_PaGenBase.png]

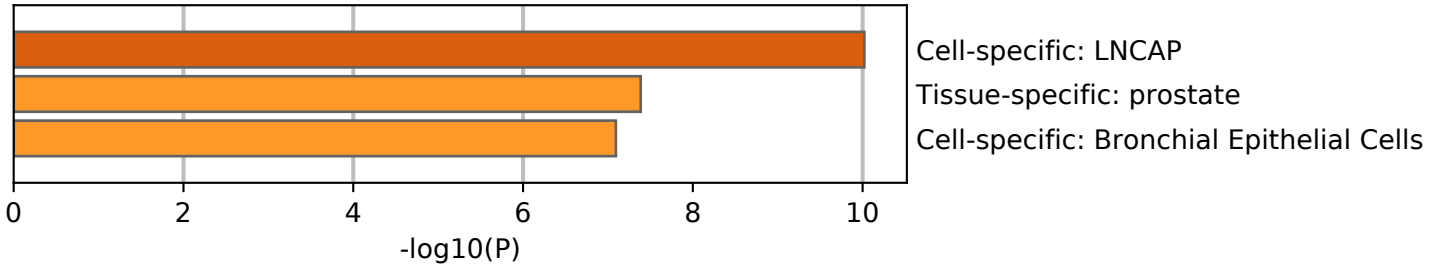

Supplement: Supplementary file 1 [file genes-11-01493-s001.zip › Enrichment_QC/HeatmapSelectedGO_PaGenBase.pdf]

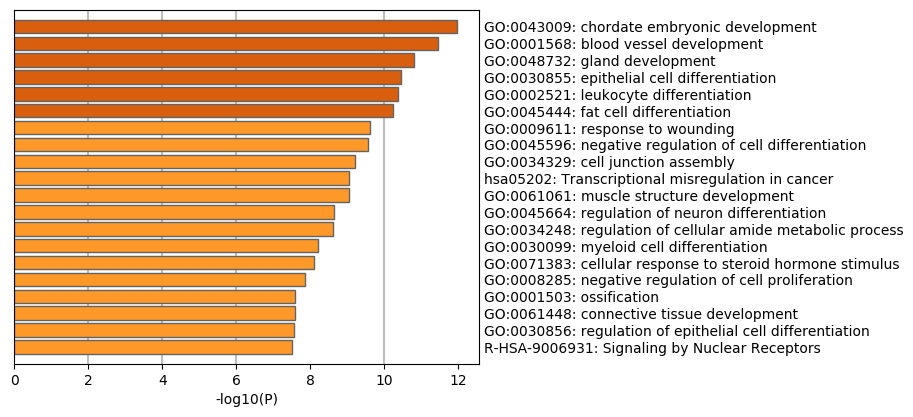

Supplement: Supplementary file 1 [file genes-11-01493-s001.zip › Enrichment_heatmap/HeatmapSelectedGO.png]

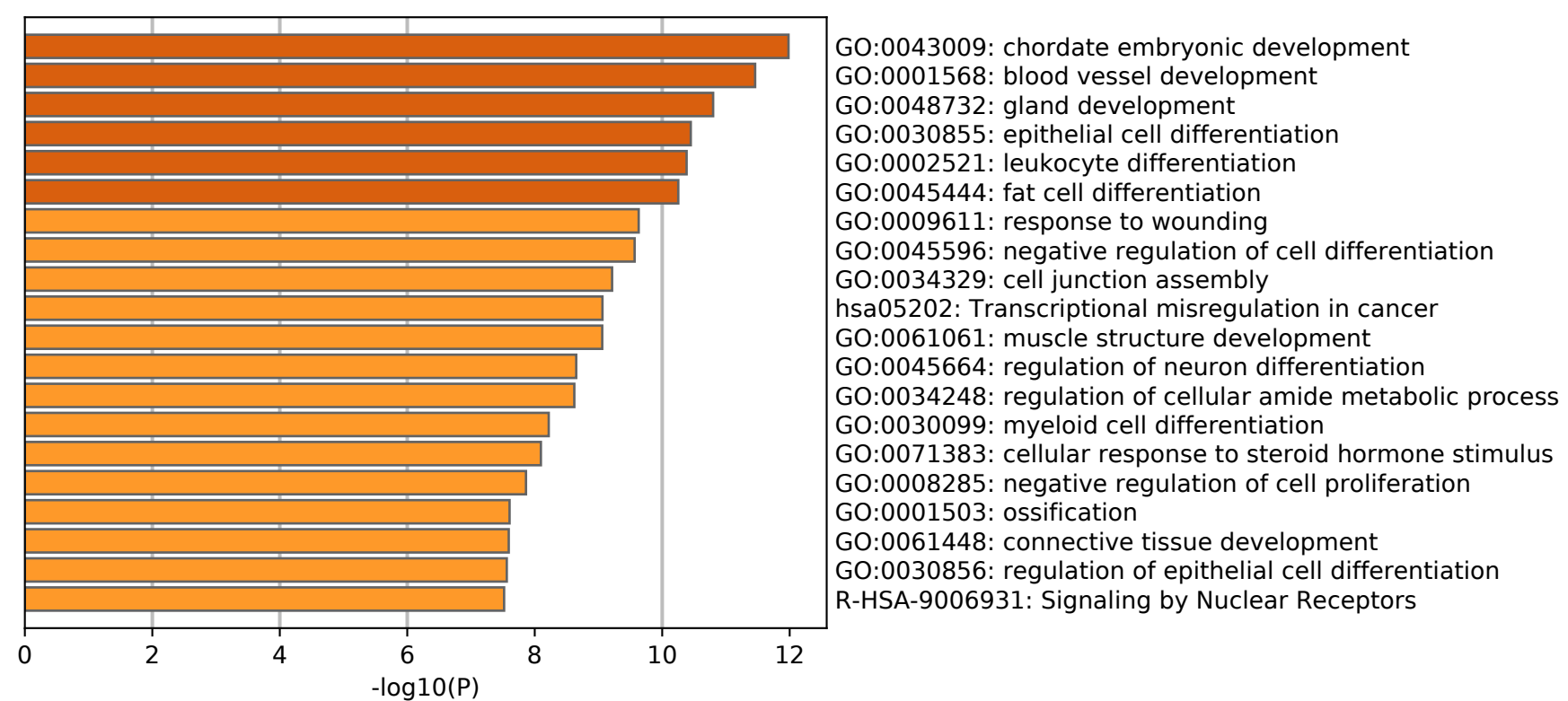

Supplement: Supplementary file 1 [file genes-11-01493-s001.zip › Enrichment_heatmap/HeatmapSelectedGO.pdf]

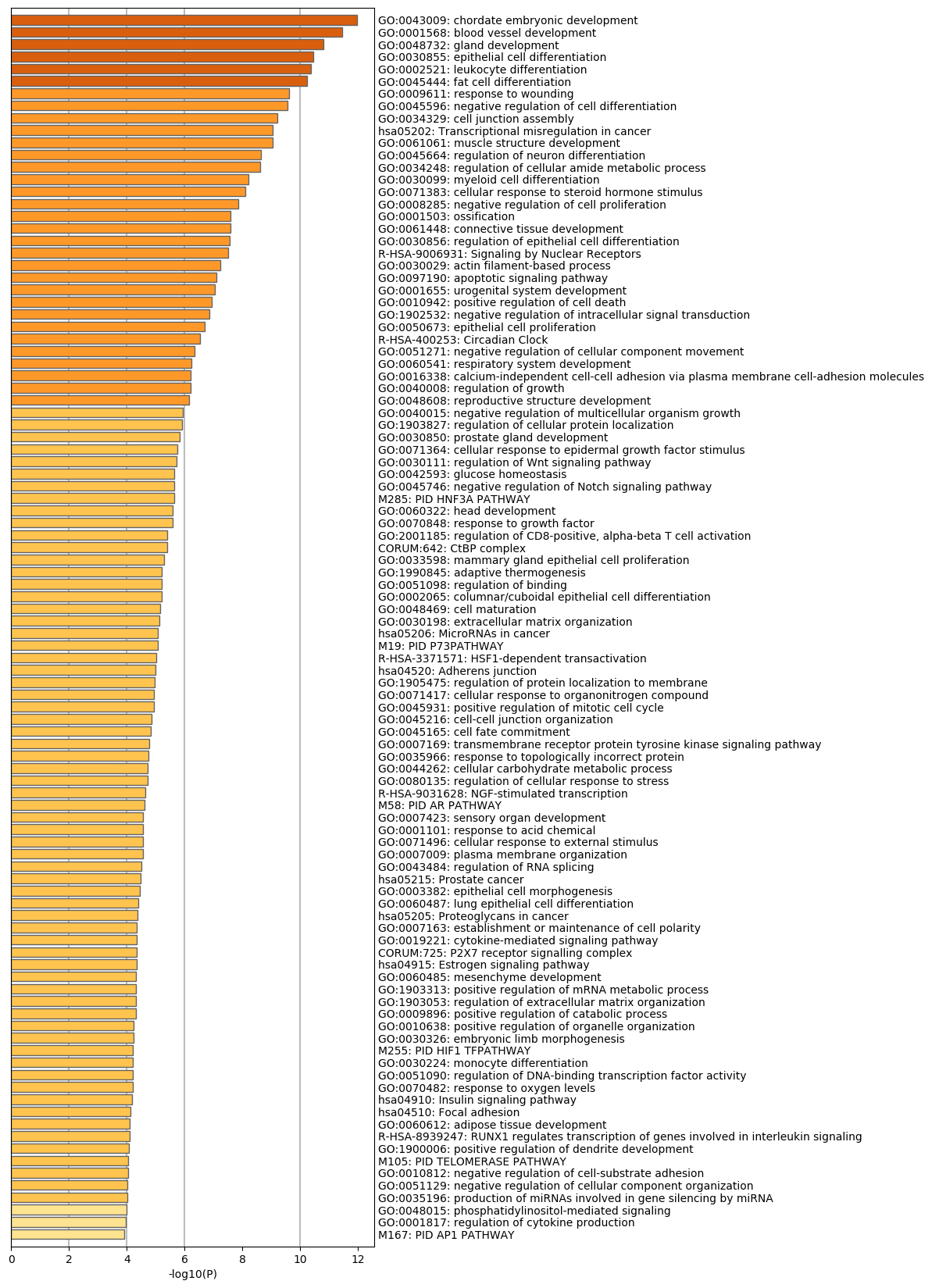

Supplement: Supplementary file 1 [file genes-11-01493-s001.zip › Enrichment_heatmap/HeatmapSelectedGOTop100.png]

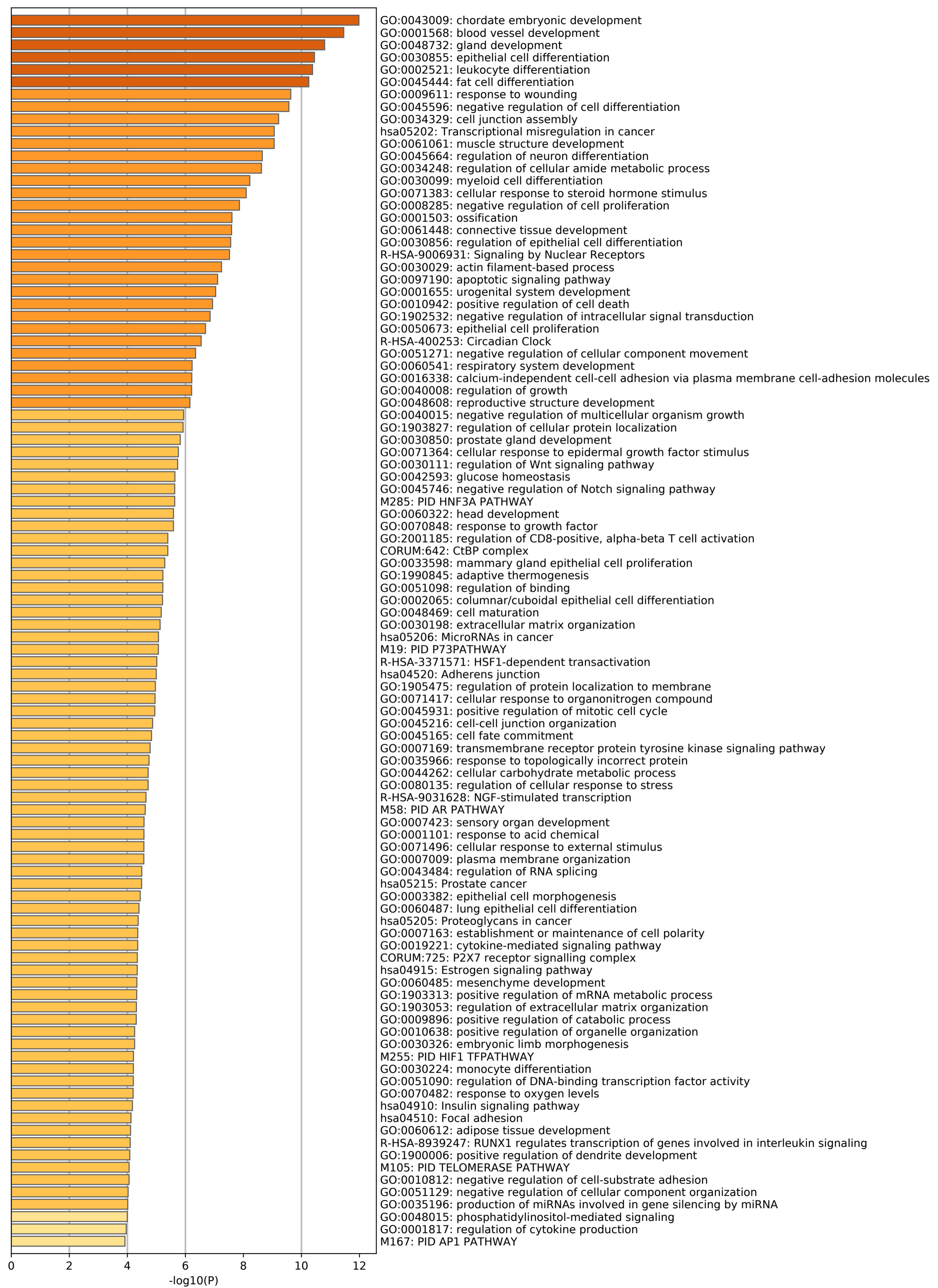

Supplement: Supplementary file 1 [file genes-11-01493-s001.zip › Enrichment_heatmap/HeatmapSelectedGOTop100.pdf]

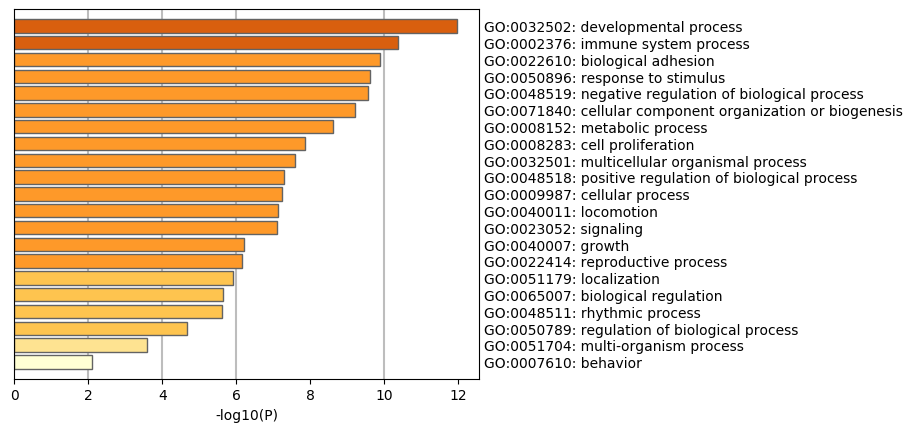

Supplement: Supplementary file 1 [file genes-11-01493-s001.zip › Enrichment_heatmap/HeatmapSelectedGOParent.png]

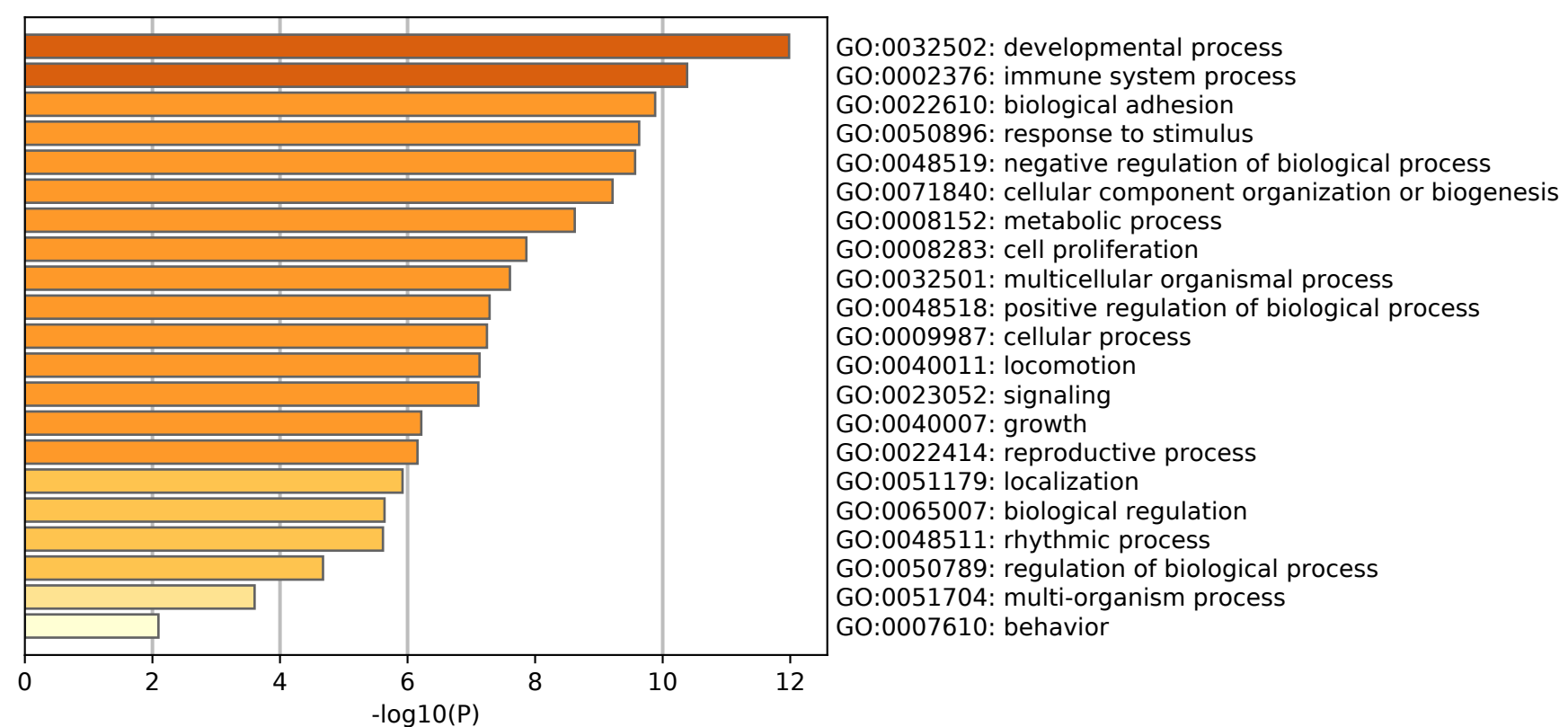

Supplement: Supplementary file 1 [file genes-11-01493-s001.zip › Enrichment_heatmap/HeatmapSelectedGOParent.pdf]
